# A possible cause of testicular cancer

**DOI:** 10.1054/bjoc.1999.1137

**Published:** 2000-05-18

**Authors:** W H James

**Affiliations:** The Galton Laboratory, University College London, Wolfson House, 4 Stephenson Way, London, NW1 2HE, UK


and Stenning, 1997). Identification of patients at an earlier stage, prior to induction therapy may allow patients predicted to do badly with cisplatin/ifosfamide based therapy to receive alternative experimental approaches.

*J Shamash, RTD Oliver, CJ Gallagher, AC Newland, TA Lister,*
*S Kelsey, RK Gupta and CA O'Doherty*
*St Bartholomew's Hospital, Theatre D, 1st Floor*
*King George V Building, West Smithfield, London EC1A 7BE, UK*

**REFERENCES**

Beyer J, Kramar A, Mandanas R, Linkesch W, Greinix A, Droz JP, et al (1996) High-dose chemotherapy as salvage treatment in germ cell tumors: a multivariate analysis of prognostic variables. *J Clin Oncol* **14**: 2638–2645

Calvert AH, Newell DR and Gumbrell LA (1984) Carboplatin dosage: prospective evaluation of a simple formula based on renal function. *J Clin Oncol* **7**: 1748–1756

Mead GM and Stenning S (1997) International germ cell cancer collaborative group prognostic factor scoring system. *J Clin Oncol* **15**: 594–603

doi: 10.1054/ bjoc.1999.1137, available online at http://www.idealibrary.com on IDEAL®

### A possible cause of testicular cancer

**Sir**

Fosså and Kravdal (2000) found that men with testicular cancer were subfertile both before and after diagnosis. Møller (1998) reported that men with testicular cancer sired significant excesses of daughters both before and after diagnosis. Fosså and Kravdal (2000) wrote that 'the low fertility after diagnosis may be partly due to the continuing inherent influence of a sub- or in-fecundity that also had a bearing on the development of the disease'.

Any cause of both the subfertility and the low offspring sex ratio (proportion male) would therefore seem a promising candidate also as a cause of the disease, so I should like to suggest one.

I have adduced very substantial quantities of data to support the hypothesis that the sexes of mammalian (and among them, human) offspring are partially dependent on the hormone levels of both parents around the time of conception (James, 1996). *Ex hypothesi*, low androgen levels in either parent are associated with the subsequent production of daughters. Moreover, levels of androgens (particularly dihydrotestosterone) in men are positively associated with coital rates (Dabbs and Morris, 1990; Mantzoros et al, 1995): and coital rates are powerfully and positively associated with fecundability, the probability of conceiving in a month at risk (Potter and Millman, 1986). Accordingly, I suggest that one cause of testicular cancer is suboptimal androgen levels. These, in turn, may be influenced by the intrauterine environment in which the patient gestated.

*WH James*
*The Galton Laboratory, University College London,*
*Wolfson House, 4 Stephenson Way, London NW1 2HE, UK*

**REFERENCES**

Dabbs JM and Morris R (1990) Testosterone, social class and antisocial behavior in a sample of 4462 men. *Psychol Sci* **1**: 1–3

Fosså SD and Kravdal Ø (2000) Fertility in Norwegian testicular cancer patients. *Br J Cancer* **82**: 737–741

James WH (1996) Evidence that mammalian sex ratios at birth are partially controlled by parental hormone levels at the time of conception. *J Theoret Biol* **180**: 271–286

Mantzoros CS, Georgiadis EI and Trichopoulos D (1995) Contribution of dihydrotestosterone to male sexual behaviour. *Br Med J* **310**: 1289–1291

Møller H (1998) Trends in sex ratio, testicular cancer and male reproductive hazards: are they connected? *Acta Pathol Microbiol Scand* **106**: 232–239

Potter RG and Millman SR (1986) Fecundability and the frequency of marital intercourse: new models incorporating the aging of gametes. *Population Stud* **40**: 159–170.

*© 2000 Cancer Research Campaign*

